# Therapy delay due to COVID-19 pandemic among European women with breast cancer: prevalence and associated factors

**DOI:** 10.1007/s00432-023-05065-7

**Published:** 2023-07-05

**Authors:** Niklas Gremke, Sebastian Griewing, Elena Bausch, Svetlana Alymova, Uwe Wagner, Karel Kostev, Matthias Kalder

**Affiliations:** 1 Department of Gynecology and Obstetrics, University Hospital Marburg, Philipps-University Marburg, Baldingerstraße, 35043 Marburg, Germany; 2 Institute of Molecular Oncology, Philipps-University Marburg, Hans-Meerwein-Straße 3, 35043 Marburg, Germany; 3 Real World Solutions, IQVIA, Unterschweinstiege 2–14, 60549 Frankfurt, Germany; 4 IQVIA, Unterschweinstiege 2–14, 60549 Frankfurt, Germany

**Keywords:** Breast Cancer, COVID-19 pandemic, Therapy delay

## Abstract

**Purpose:**

This study investigates the impact of the COVID-19 pandemic on breast cancer (BC) care, analyzing treatment delays and factors associated with them.

**Methods:**

This retrospective cross-sectional study analyzed data from the Oncology Dynamics (OD) database. Surveys of 26,933 women with BC performed between January 2021 and December 2022 in Germany, France, Italy, the United Kingdom, and Spain were examined. The study focused on determining the prevalence of treatment delays due to the COVID-19 pandemic, considering factors such as country, age group, treating facility, hormone receptor status, tumor stage, site of metastases, and Eastern Cooperative Oncology Group (ECOG) status. Baseline and clinical characteristics were compared for patients with and without therapy delay using chi-squared tests, and a multivariable logistic regression analysis was conducted to explore the association between demographic and clinical variables and therapy delay.

**Results:**

The present study found that most therapy delays lasted less than 3 months (2.4%). Factors associated with higher risk of delay included being bedridden (OR 3.62; 95% CI 2.51–5.21), receiving neoadjuvant therapy (OR 1.79; 95% CI 1.43–2.24) compared to adjuvant therapy, being treated in Italy (OR 1.58; 95% CI 1.17–2.15) compared to Germany or treatment in general hospitals and non-academic cancer facilities (OR 1.66, 95% CI 1.13–2.44 and OR 1.54; 95% CI 1.14–2.09, respectively) compared to treatment by office-based physicians.

**Conclusion:**

Addressing factors associated with therapy delays, such as patient performance status, treatment settings, and geographic location, can help guide strategies for improved BC care delivery in the future.

## Introduction

The global outbreak of the coronavirus disease (COVID-19) has had a significant and far-reaching effect on cancer treatment and management worldwide (Papautsky and Hamlish 2020). In particular, health systems have shown notable differences in their resilience and organizational structures, with the outbreak of the pandemic revealing vulnerabilities in terms of managing resources for the care of COVID-19 patients (Griewing et al. 2022; van de Haar et al. 2020). In many countries, health authorities advised hospitals and health care facilities to defer medical care for non-acute or non-life-threatening conditions and postpone cancer screenings to minimize the number of hospital visits and save resources and intensive care capacities (Arndt et al. 2023; Erdmann et al. 2021; Gremke et al. 2022). Consequently, there was a notable deceleration in all facets of breast cancer (BC) care, including diagnostic procedures and treatments (chemotherapy and radiation therapy), routine follow-up, psycho-oncology care, and genetic counseling (Lang et al. 2023). For example, Gasparri and colleagues conducted a survey of 377 European breast centers in 41 countries demonstrating that the estimated time interval between BC diagnosis and treatment initiation increased for about 20% of the participating institutions during the pandemic. Notably, modifications in primary systemic therapy indications were reported by 56% of respondents (211/377). Rates of upfront surgery increased from 39.8 to 50.7% (*p* < 0.002) in patients with T1cN0 triple-negative BC and from 33.7 to 42.2% (*p* < 0.016) in ER-negative/HER2-positive cases (Gasparri et al. 2020). While it is not clear how deviations from the standard of care will affect long-term outcomes in BC patients (e.g., increased use of neoadjuvant endocrine therapy), it is well known that delays between BC diagnosis and treatment initiation have been linked to poorer survival outcomes in BC patients (Satish et al. 2021). Importantly, a prospective analysis showed that a delay in starting chemotherapy (defined as a gap of over 8 weeks from diagnosis or prior treatment) was associated with a higher risk of BC-specific mortality (BCSM) (HR = 1.71; 95% CI 1.07–2.75) and all-cause mortality (ACM) (HR = 1.39; 95% CI 1.02–1.90). A delay in radiation therapy increased BCSM risk (HR = 1.49; 95% CI 1.00–2.21) but not ACM risk (HR = 1.19; 95% CI 0.99–1.42) (Yung et al. 2020).

Analyzing the underlying factors associated with treatment delay in BC patients is thus important in order to prevent suboptimal treatment and improve preparedness for future COVID-variants, pandemics or other major disruptions to health care. Here, we present the findings of a retrospective cross-sectional study involving 26,933 patients from five European countries. The aim of the study is to analyze the occurrence of treatment delays caused by the COVID-19 pandemic, taking into account various factors (e.g., age group, treating facility, tumor stage, ECOG status).

## Methods

### Database

This retrospective cross-sectional study is based on data from IQVIA’s Oncology Dynamics (OD) database (Alymova et al. 2022; Kadys et al. 2023). This source is supplied with data by means of cross-sectional partially retrospective surveys collecting anonymized patient information from a representative panel of physicians involved in pharmacological cancer treatment. The OD collects fully anonymized patient-level data on drug-treated cancer cases in several countries worldwide. Data collection and reporting is conducted through a standardized online questionnaire in which all items are mandatory. A reporting manual with precise instructions on completing the questionnaire is provided to each respondent. Specific instructions are displayed by means of a ‘pop-up’ system throughout the survey to provide clear definitions for the desired variables. Physicians are also asked to enter factual information from patient medical records to avoid recall bias. Further tactics to ensure input accuracy include controlled code lists and multiple-choice questions, as well as interactive filters that limit non-applicable questions (e.g., items on cancer-specific biomarkers). Responses are immediately validated against previous answers and reference files; “unexpected value” messages are displayed to the participant in the case of anomalies, prompting them to double-check their response. Physicians are instructed to report the most recent consecutive cases (up to 20 cases depending on the specialty) they have treated during the last observation period to discourage selective case submission. After the form is submitted, a number of additional validations and trend checks are performed; anomalous values are discussed with the submitting participant and corrected as needed.

### Patient selection, study outcome, and variables

Surveys of all women with breast cancer diagnoses made between January 1st 2021 and December 31st 2022 were available for five European countries: Germany, France, Italy, the United Kingdom (UK), and Spain. The main outcome of the study was the prevalence of treatment delays due to the COVID-19 pandemic depending on different factors including country, age group, treating facility (academic cancer facility, non-academic cancer facility, general hospital, office-based practitioner), hormone receptor status (estrogen and progesterone receptor), current tumor stage, site of metastases (liver, lung, bones), and ECOG performance status (asymptomatic, symptomatic, fully ambulatory, symptomatic in bed less than 50%, symptomatic in bed more than 50%, and bedridden). Information on therapy delay was distinguished into three categories: postponed by less than 3 months, 3–6 months, and > 6 months.

### Statistical analysis

First, baseline and clinical characteristics were compared for patients with and without therapy delay using Chi-squared tests. Then, a multivariable logistic regression model was used to investigate the association between demographic and clinical variables and therapy delay (yes versus no). *P*-values < 0.05 were considered statistically significant. All analyses were performed using SAS 9.4 (SAS Institute, Cary, US).

## Results

### Baseline characteristics of study population

Overall, 26,933 patients from 5 different European countries were included. Baseline characteristics of the study population are shown in Table 1. Patients were 62.0 (SD: 13.2) years old on average. Of the total number of patients included in the study, 24.5% were treated in Germany, 21.0% in France, 14.1% in the UK, 18.3% in Spain, and 21.8% in Italy. Overall, 36.5% of patients were treated in academic cancer facilities, 27.5% in non-academic cancer facilities, 5.7% in general hospitals, and 16.1% by office-based physicians. The majority (66.4%) were both PR- and ER-positive; 20.9% were PR and ER negative. Most patients were either asymptomatic (49.8%) or symptomatic but fully ambulatory (41.2%) according to their ECOG status. First-line advanced/metastatic therapy (32.8%) and adjuvant therapy (42.8%) were most common in the study population.

**Table 1:** Baseline characteristics of study patients based on surveys of all women with breast cancer diagnoses between January 1st 2021 and December 31st 2022

Variable	Total
*N*	26,933
Age (mean, SD)	62.0 (13.2)
*Age group (N,%)*	
≤ 40	1432 (5.3)
41–50	4298 (16.0)
51–60	6233 (23.1)
61–70	7559 (28.1)
> 70	7411 (27.5)
*Country*	
France	5665 (21.0)
Germany	6668 (24.5)
Italy	5875 (21.8)
Spain	4925 (18.3)
UK	3800 (14.1)
*Treating facility*	
Academic cancer facility	9833 (36.5)
Non-academic cancer facility	7393 (27.5)
General hospital	1545 (5.7)
Office-based practitioner	4327 (16.1)
Unknown	3835 (14.2)
*Hormone status*	
PR-positive	479 (1.8)
ER-positive	2421 (9.0)
Both PR- and ER-positive	17882 (66.4)
Neither PR- nor ER-positive	5636 (20.9)
Unknown	515 (1.9)
*Current stage grade*	
Localized	11,145 (41.4)
Locally advanced	3297 (12.2)
Advanced	426 (1.6)
Metastatic	12,065 (44.8)
*Site of distant metastasis (most frequent)*	
Bones	7184 (26.7)
Liver	3890 (14.4)
Lung	4152 (15.4)
Brain	577 (2.1)
Skin	1074 (4.0)
*ECOG performance status*	
Asymptomatic	13,421 (49.8)
Symptomatic fully ambulatory	11,103 (41.2)
Symptomatic in bed less than 50%	2146 (8.0)
Symptomatic in bed more than 50%	222 (0.8)
Bedridden	30 (0.15)
*Current line of therapy*	
1st line advanced/metastatic	8824 (32.8)
2nd line advanced/metastatic	1991 (7.4)
3rd line advanced/metastatic	802 (3.0)
4th line advanced/metastatic	684 (2.5)
Adjuvant	11,409 (42.4)
Neo-adjuvant	2859 (10.6)
Early-stage/primary therapy	364 (1.4)

### Prevalence of treatment delay

**Fig. 1:**
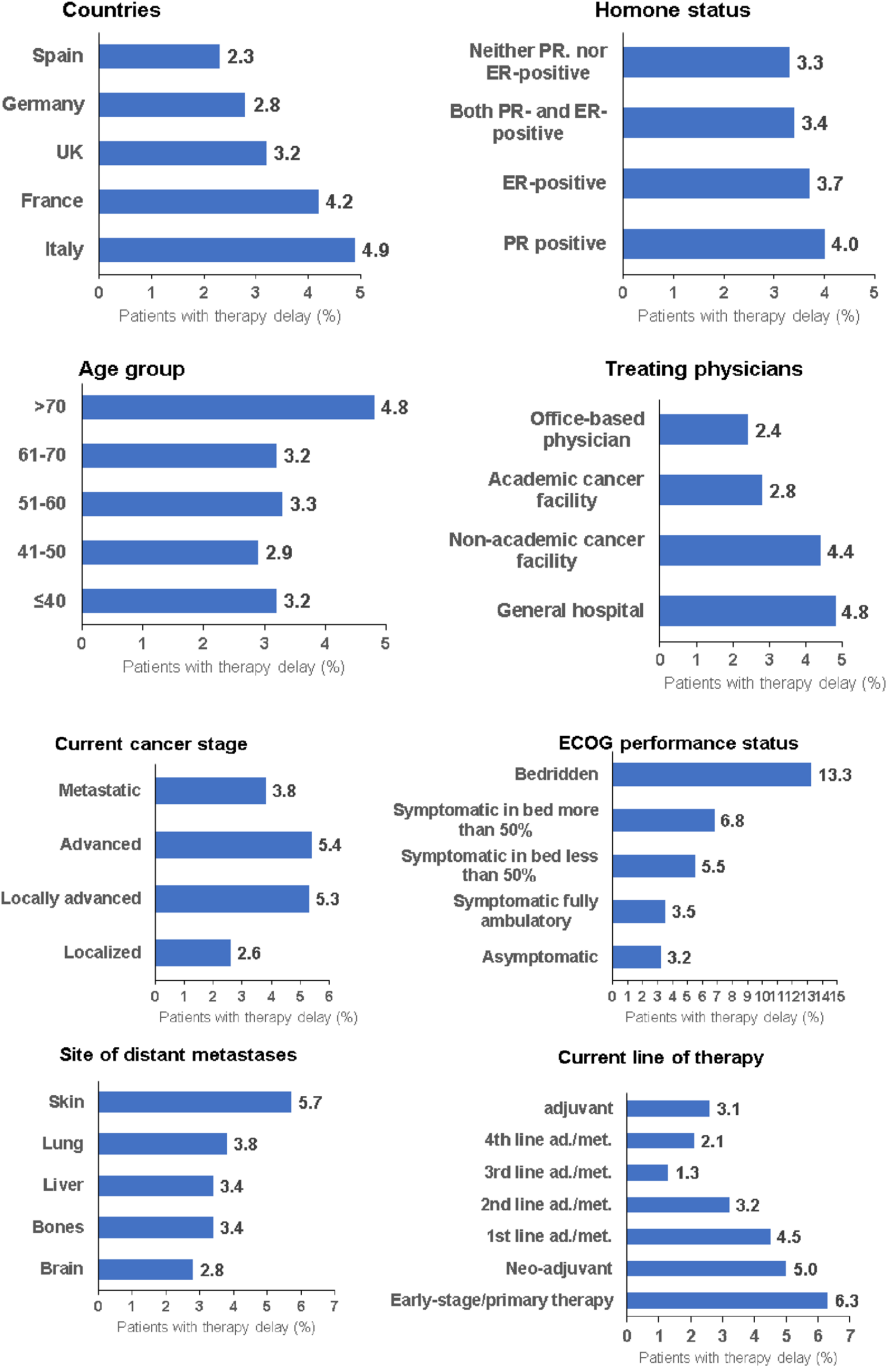
Prevalence of therapy delay by country, hormone status, age group, treating physician, current cancer stage, ECOG performance status, site of metastases and current line of therapy

Some degree of treatment delay was documented in 947 (3.5%) study patients. When treatment delay occurred, therapy was usually postponed by less than 3 months (2.4%), followed by 3–6 months (0.6%). Figure 1 shows the prevalence of treatment delay depending on different variables. The biggest difference was observed between asymptomatic and bedridden women (3.2% versus 13.3%, *p* < 0.001). Furthermore, the prevalence of therapy delay was higher in Italy (4.9%) and France (4.2%) than in Germany (2.8%) and Spain (2.3%) (*p* < 0.001). Delays were more common in general hospitals (4.8%) and non-academic cancer facilities (4.4%) than in academic facilities (2.8%) and in patients treated by office-based physicians (2.4%). Among patients receiving early-stage/primary therapy, 6.3% experienced therapy delay compared to 2.1% of patients in 4th line metastatic/advanced therapy (*p* < 0.001).

### Results of multivariable regression model

Table 2 shows the results of the multivariable logistic regression for the association between predefined variables and therapy delay. The strongest positive association with therapy delay was observed for bedridden patients (OR 3.62; 95% CI 2.51–5.21) versus asymptomatic patients. 1st line advanced/metastatic therapy (OR 2.16; 95% CI 1.42–3.28), early-stage/primary therapy (OR 1.92; 95% CI 1.22–3.01), and neo-adjuvant therapy (OR 1.79; 95% CI 1.43–2.24) were associated with a higher risk of therapy delay than adjuvant therapy. Locally advanced cancer was more strongly associated with therapy delay (OR 2.05; 95% CI 1.34–3.13) than metastatic cancer. There was also a positive association between being treated in Italy (OR 1.58; 95% CI 1.17–2.15) versus Germany, and therapy in a general hospital (OR 1.66, 95% CI 1.13–2.44) or a non-academic cancer facility (OR 1.54; 95% CI 1.14–2.09) versus office-based physicians in regards to therapy delay risk.

**Table 2:** Association between demographic and clinical variables and therapy delay among women with breast cancer

Variable	Adjusted Odds Ratio (95% CI)	*p* value
*Age group*		
≤ 40	Reference	
41–50	0.90 (0.64–1.27)	0.544
51–60	0.98 (0.70–1.36)	0.901
61–70	0.91 (0.66–1.27)	0.588
> 70	1.12 (0.81–1.57)	0.491
*Country*		
France	1.23 (0.95–1.61)	0.120
Germany	Reference	
Italy	**1.58 (1.17–2.15)**	**0.003**
Spain	0.79 (0.58–1.06)	0.114
UK	0.83 (0.62–1.13)	0.240
*Treating facility*		
Academic cancer facility	1.13 (0.84–1.58)	0.410
Non-academic cancer facility	**1.54 (1.14–2.09)**	**0.005**
General hospital	**1.66 (1.13–2.44)**	**0.010**
Office-based practitioner	Reference	
*Hormone status*		
PR-positive	1.41 (0.86–2.30)	0.174
ER-positive	1.24 (0.95–1.61)	0.110
Both PR- and ER-positive	1.13 (0.94–1.36)	0.180
Neither PR- nor ER-positive	Reference	
*Current stage*		
Localized	1.19 (0.75–1.88)	0.465
Locally advanced	**2.05 (1.34–3.13)**	**0.001**
Advanced	1.66 (1.00–2.79)	0.052
Metastatic	Reference	
*Site of distant metastasis (most frequent)*		
Bones	0.82 (0.67–1.01)	0.056
Liver	0.95 (0.76–1.18)	0.625
Lung	1.08 (0.87–1.33)	0.496
Brain	0.81 (0.48–1.36)	0.433
Skin	1.32 (0.99–1.76)	0.058
*ECOG performance status*		
Asymptomatic	Reference	
Symptomatic fully ambulatory	1.38 (0.84–2.25)	0.202
Symptomatic in bed less than 50%	1.21 (0.93–1.58)	0.152
Symptomatic in bed more than 50%	1.11 (0.92–1.33)	0.281
Bedridden	**3.62 (2.51–5.21)**	** < 0.001**
*Current line of therapy*		
1st line advanced/metastatic	**2.16 (1.42–3.28)**	** < 0.001**
2nd line advanced/metastatic	1.58 (0.98–2.55)	0.060
3rd line advanced/metastatic	0.62 (0.29–1.33)	0.220
4th line advanced/metastatic	0.94 (047–1.86)	0.852
Adjuvant	Reference	
Neo-adjuvant	**1.79 (1.43–2.24)**	** < 0.001**
Early-stage/primary therapy	**1.92 (1.22–3.01)**	**0.005**

Significant results are in bold

## Discussion

In this large retrospective cross-sectional study including 26,933 BC patients from five European countries, we identified multiple factors associated with treatment delays during the COVID-19 pandemic (Dietz et al. 2020; Ueda et al. 2020). Importantly, we found that patient performance status, treatment setting, and geographic location were associated with delays in BC treatment during the pandemic. Our study therefore offers valuable insights into the state of BC care during the most notable public health crisis of our time.

With the onset of the COVID-19 pandemic in 2020, physicians and healthcare systems faced significant challenges and uncertainties. Many were unprepared for the global outbreak of an infectious disease and lacked contingency plans. Consequently, various unstandardized adaptation strategies were swiftly implemented to prioritize high-risk patients and modify or redistribute healthcare services (Mullangi et al. 2022). A systematic review identified various determinants for delays and disruptions in cancer health care due to the COVID-19 pandemic, with reductions in routine cancer services and the number of cancer surgeries, delays in radiation therapy, and delays or cancellations of outpatient visits being the most common causes (Riera et al. 2021). Notably, all of these factors delaying cancer treatment can generally be grouped into patient-related (e.g., travel difficulties due to lockdown or immobility) and healthcare-related factors (e.g., shortage of personal protective equipment and personnel, delays in surgery) (Kumar & Dey 2020). As an important patient-related factor, our study showed that therapy delay was most strongly associated with bedridden patients and least strongly associated with asymptomatic patients (OR 3.62; 95% CI 2.51–5.21). This result is in line with several studies showing that individuals with disabilities and multiple comorbidities experienced more significant delays in receiving care during the pandemic (Czeisler et al. 2020; Lang et al. 2023; Mullangi et al. 2022). For example, a prospective cohort study from the U.S. revealed that therapy delays were greater among multimorbid patients (OR 1.23; 95% CI 1.00–1.53). Particularly compared with patients with 0 to 1 comorbidities, having 2 or more comorbidities was more strongly associated with chemotherapy delay (OR 1.23; 95% CI 1.00–1.53) as well as radiation therapy delay (OR 2.69; 95% CI 1.20–6.20) (Mullangi et al. 2022). To summarize, this particular group of patients faced a dual impact from the pandemic. They not only had a heightened vulnerability to severe COVID-19 infection, but were also more likely to experience delays in receiving medical care due to their more acute medical requirements (Richards et al. 2020). In addition, our findings show that 1st line advanced/metastatic therapy (OR 2.16; 95% CI 1.42–3.28), early-stage/primary therapy (OR 1.92; 95% CI 1.22–3.01), and neo-adjuvant therapy (OR 1.79; 95% CI 1.43–2.24) were associated with a higher risk of therapy delay than adjuvant therapy. When these results are considered in the context of current literature, one notable distinction is the increased utilization of neoadjuvant systemic therapy as a result of the postponement of numerous breast surgeries during the pandemic (Dietz et al. 2020; Escobar et al. 2023; Sheng et al. 2020). It is all the more unfavorable if, for example, there are delays in neoadjuvant chemotherapy due to the absence of telemedicine structures, a lack of human and material resources in oncology practices and patients’ fear of possible Covid infection (Caston et al. 2021). Of course, the same factors can also be responsible for the therapy delays in 1st line treatment for patients with metastatic BC disease and primary therapy in those with early-stage BC mentioned above. However, it must be added that recommendations for patients with stable metastatic BC proposed by the COVID-19 Pandemic Breast Cancer Consortium advise a Priority C categorization, meaning that certain treatments or services can be deferred indefinitely until the pandemic is over without adversely impacting outcomes, which may explain the observed therapy delay for 1st line advanced/metastatic therapy (OR 2.16; 95% CI 1.42–3.28) (Dietz et al. 2020). Additionally, in the context of early-stage BC, the therapy delays described (OR 1.92; 95% CI 1.22–3.01) are also justifiable based on recommendations from the COVID-19 Pandemic Breast Cancer Consortium. For instance, for patients aged 65–70 years or older with early-stage, node-negative, estrogen receptor-positive invasive disease, the advice is to initiate endocrine therapy after surgery, while radiation therapy (RT) can be safely postponed or omitted until the pandemic subsides (Dietz et al. 2020; Hughes et al. 2013; Kunkler et al. 2015).

Next, our findings also show that the frequency of delays in cancer treatment was found to be higher in general hospitals (4.8%) and non-academic cancer facilities (4.4%) than in academic facilities (2.8%) and office-based physicians (2.4%). One study by Papautsky et al. surveyed 609 adult BC survivors in the U.S. to determine whether they had experienced delays in cancer-related care or treatment. In particular, the study compared demographic and clinical characteristics as well as the site of care for those who reported delays and those who did not. Interestingly, the lowest frequency of delays was found in cancer centers (35%, *p* = 0.13) and, in line with our data, the highest frequency (albeit not statistically significant) was observed in Veterans Affairs hospitals (50%, *p* = 0.76) and community hospitals (*p* = 0.45) (Papautsky and Hamlish 2020).

There was also a positive association between therapy delay and treatment in Italy (OR 1.58; 95% CI 1.17–2.15) compared to Germany. Notably, Italy was the first European country to be severely impacted by the COVID-19 pandemic, placing extraordinary pressure on the country's healthcare and long-term care systems (Mangone et al. 2022; Parotto et al. 2022; Toss et al. 2021). Within a month of the first reported case in the Lombardy region in northern Italy on April 1, the country had already reported over 100,000 cases and more than 12,000 deaths (Fratino et al. 2020; Madan et al. 2020). In this regard, a multicentric retrospective study analyzed data from four Italian BC units focusing on the delay in BC treatments during the first COVID-19 lockdown. This study by Vanni and colleagues revealed that the time span between breast biopsy and surgery was shorter in the pre-lockdown group than in the lockdown group. The mean values were 42 days versus 56 days respectively, and the difference between the groups was statistically significant (Vanni et al. 2020). The authors concluded that these delays were caused by the slowdown in oncological treatments during the lockdown, stemming from health system reorganization and resource reallocation (Buonomo et al. 2020). To the best of our knowledge no studies to date have made international comparisons between European countries regarding therapy delays in BC patients during the COVID pandemic making ours first to reveal this international difference.

Finally, we analyzed the prevalence and duration of treatment delays and found that 3.5% of the study population had experienced therapy delays, which typically lasted less than 3 months (2.4%). When considering these data in relation to the existing body of literature, we noticed that there is substantial variation with respect to the reported frequency and duration of therapy delays. In particular, a systematic review focusing on delays and disruptions in cancer healthcare due to the COVID-19 pandemic, mainly from surveys and cross-sectional studies, revealed that a delay in cancer treatment was observed in 5–52.6% of patients (Bogani et al. 2020; Riera et al. 2021). Stratified by therapy type, a delay in surgery was reported by 3.34–76%, a delay in radiotherapy by 1.4–90.9% (Achard et al. 2020; Anacak et al. 2020; Thaler et al. 2020), and a delay in chemotherapy by 6.3–20.3% (Riera et al. 2021; Thaler et al. 2020) of patients, physicians, and centers. However, it is important to note that a direct 1:1 comparison between our data and the data from these studies is not feasible due to variations in factors such as country-specific healthcare systems, policies, cancer types, and the specific stages of the pandemic during which the data were collected (Riera et al. 2021).

In conclusion, our study provides important insights into the factors associated with treatment delays in BC patients during the COVID-19 pandemic and highlights the need for tailored strategies to minimize delays and ensure timely and effective care for BC patients. Further research is needed to address these challenges and optimize BC cancer care delivery in the event of future public health crises.

## Strengths and limitations

The main advantage of this study is the unique database used, which includes data pertaining to a significant number of patients from various countries. This allows for the investigation and comparison of differences among BC patients on an international scale. Moreover, the present set of analyses demonstrates an overall agreement between the OD database, published epidemiological literature, and public data sources (Alymova et al. 2022). However, our study is subject to certain limitations that should be acknowledged at this point. The original questionnaire used was not intended for research purposes, resulting in the absence of variables such as behavioral risk factors and socioeconomic status. It is worth noting that the database only includes patients receiving drug treatment and patients included were predominantly treated by oncologists (Alymova et al. 2022). Therefore, it is plausible that individuals with localized-stage BC might have a lower likelihood of being included in the study, potentially leading to a higher proportion of Stage III and IV BC patients compared to the overall BC patient population. Additionally, there is a potential selection bias due to the recruitment of participants through selective healthcare providers rather than a population-based approach for example in terms of stage at diagnosis and treatment. Finally, the present study design does not allow causal relationships to be estimated, only associations.

## Data Availability

Anonymized raw data are available upon reasonable request.
